# Editorial: Motivation in learning and performance in the arts and sports

**DOI:** 10.3389/fpsyg.2025.1554109

**Published:** 2025-02-10

**Authors:** Adina Mornell, Margaret S. Osborne, Noa Kageyama, Frank Heuser

**Affiliations:** ^1^University of Music and Theater Munich, Munich, Germany; ^2^Melbourne School of Psychological Sciences and Melbourne Conservatorium of Music, University of Melbourne, Melbourne, VIC, Australia; ^3^The Juilliard School, New York, NY, United States; ^4^Cleveland Institute of Music, Cleveland, OH, United States; ^5^The UCLA Herb Alpert School of Music, University of California, Los Angeles, Los Angeles, CA, United States

**Keywords:** motivation, music, performance science, pedagogy, cognitive reappraisal, wellbeing

## Introduction

Motivation is a crucial factor in achieving success in any field, but especially in the performing arts and sports, where consistent practice, discipline, and creativity are required to maintain a high level of performance. The science of motivation is relevant today more than ever for several reasons. First, motivation is a fundamental aspect of human behavior, and understanding what drives individuals to perform at their best is essential in many contexts, including education, athletics, and the workplace. Second, motivation is critical for achieving goals, and an understanding of what motivates individuals can help them to set and achieve realistic and meaningful aims. Third, motivation is essential for maintaining mental and physical health and wellbeing, and an exploration of contributory factors can help individuals manage stress and anxiety.

This Research Topic collected articles that provide insights into motivation and drive over the short and long term, even in the face of challenges that may arise. The Research Topic provides interdisciplinary perspectives on motivation and explores factors that motivate learners and professionals to optimize their training methods in order to sustain effort over time. The editors hope that this Research Topic will aid in the development of strategies that promote and maintain motivation.

## Summaries

Wieser et al.: *Who stays? Who goes? Motivational Development and Tendency to drop out in Music Schools*

Two articles in this Research Topic investigate the contribution of basic psychological needs (BPN) satisfaction to optimal emotional and behavioral outcomes across music and sport at different stages of expertise. In the first, Wieser et al., explore the factors contributing to music student dropout. Autonomy, competence, and social relatedness need satisfaction were considered alongside parental involvement as key variables in shaping students' motivation and ultimately their decision to stay or leave music school. Their survey of 140 Austrian music students between the ages of 8 and 27 (*M*_age_ = 12.86, SD = 3.89) reveals that BPN satisfaction during lessons, coupled with parental involvement, significantly predicts autonomous (self-determined) motivation, which in turn was strongly associated with persistence in music school. Conversely, controlled motivation, driven by external pressures or feelings of guilt, was more likely to lead to dropout. Their study emphasizes the practical implications of fostering need satisfaction and parental involvement to promote autonomous motivation and reduce dropout rates in music education.

Robazza et al.: *Athletes' basic psychological needs and emotions: the role of cognitive reappraisal*

The second BPN article, Robazza et al., presents a questionnaire study conducted with 424 competitive athletes across multiple sports. The authors investigated the influence of cognitive reappraisal and expressive suppression in connection with athletes' feelings of competence, autonomy, and relatedness, and their experiences of emotions including happiness, excitement, anxiety, dejection, and anger. Athletes with greater BPN satisfaction were more likely to utilize cognitive reappraisal as an emotion regulation strategy, which leads to more positive emotions and psychobiosocial performance states.

Immerz et al.: *Student motivation to study music and sport – a comparison between study subjects and study programs on intrinsic and extrinsic motivational aspects*

It is clear that both music and sports require highly-skilled performance based upon optimal training/practice strategies. There are often controversial discussions about the extent to which athletics and the arts are similar and dissimilar. Immerz et al., compared the motivations of music and sport majors for selecting their academic program in a questionnaire study of 151 university students in Freiburg, Germany. They found that both groups demonstrated similarly high levels of intrinsic motivation to pursue their respective field of studies, although there were differences between those enrolled in bachelor in music performance programs vs. those studying school teacher education in programs leading to state certification. Specifically, German teacher education students in both music and sport reported higher levels of extrinsic motivation compared to bachelor students. The authors suggest that this difference might stem from the more defined career paths and structured income associated with teaching compared to the less clearly defined career prospects for those pursuing non-teaching roles in music and sport.

Gumm: *Music motivation depends on what to motivate: research review of Gumm's music teaching and conducting models*

In a review of several decades of research on Gumm's models of music teaching and conducting, Gumm explores teacher and conductor behaviors, or approaches, that motivate specific learning outcomes in music education. The study contrasts two overarching priorities—“control” (e.g., where instructors provide clear task directions and corrective feedback to the student) and “release” (e.g., where students are encouraged to self-diagnose problems or rehearse with and perform for each other). While control is more prevalent, overemphasis on control can lead to teacher burnout and student dropout. Experienced educators tend to shift toward release-oriented teaching, prioritizing deeper learning and student autonomy. The author suggests that professional development could help music educators develop a balanced approach that incorporates both control and release strategies to motivate effective learning.

López-Íñiguez and McPherson: *Using a music microanalysis protocol to enhance instrumental practice*

In a multi-case study by López-Íñiguez and McPherson the authors identify and highlight a potential deficit in the traditional master-apprentice model suggest that this form of teaching does not necessarily promote self-responsibility in the learner. Indeed, developing one's self-regulatory skills is one of the most important challenges of our times (faced not only by musicians). The authors employed the “Optimal Music Practice Protocol” (OMPP) to study the learning process. This microanalysis tool offers a structure of engagement to the learner, a technique for practice that supports autonomy. In a multiple-case study, the authors followed four highly proficient cellists during the preparation of completely new repertoire for a public recital. The use of the OMPP allowed the researchers to evaluate the learners' use of the three parts of the self-regulation model (Zimmerman's SRL—see dos Santos Silva et al. above) which was informed by the self-determination theories of Deci and Ryan. These are the (1) Forethought phase, (2) Performance phase, and (3) Self-reflection phase. The study ran over a period of many months and culminated in a concert performance. In evaluating the results, the authors concluded that the OMPP is a useful framework most effective for those musicians who have a developed “learner identity.” In other words, those who adopt a mindset of life-long learning are most motivated to take the steps necessary for the learning process. They invest patience into their work, and remain humble in the face of challenges.

Du: *Modeling the predictive role of music teachers' job commitment and optimism in their sense of self-efficacy*

Music teachers' commitment and academic optimism with respect to self-efficacy were the focus of Du's questionnaire that was developed following a literature review and subsequently conducted at four universities in China. Similar to dos Santos Silva et al. conclusions (see below), the analysis of 340 participants' data led the author to suggest that teacher education in China should be augmented with psychological topics such as motivation, mental health, and support for self-competence and identity—all themes found in multiple other papers in this Research Topic. The author recommends that music teachers receive training in practical techniques to deal with the individual learners and their emotions. By widening the lens of pedagogical training, future teachers can learn to embrace skills beyond musical competence needed to best address the needs of their own students.

Satka and Garneva: *Model of motivational competence: creation of students' motivation, assessment, and research*

In their literature-based study, Satka and Garneva apply a number of analytical approaches to develop a model of motivational competence (MMC) to address their perception of decreased motivation for learning among students at all levels of education over the past several decades. From a review of motivation learning theories, they focus on “competence motivation” as put forth by Elliot and Dweck (2005). The MMC model is based on effective communication and feedback between mentor and learner, as well as the nurturing of critical thinking skills. Central to this model are competencies based on human values and virtues that co-develop during the learning process in both teachers and students. Their model suggests that educational processes emphasizing strong values and moral virtues should provide the motivation for achieving curricular purposes and academic competencies as well as high spiritual qualities and moral principles.

Latif et al.: *The impact of genre-based instruction on Saudi university students' English writing performance and motivation: a mixed-method study*

Latif et al., explore how a genre-based instructional approach, through which students learn about different types of writing, including structures, characteristics, and conventions, influence their motivation to develop their writing skills in an English writing class at a Saudi university. Using a quasi-experimental research design, student read model texts in a target genre, collaboratively wrote sample essay parts and received feedback, and independently wrote essays in the genre. Argumentative and classification writing performance improved in the areas of text content, organization, vocabulary, language use, and mechanics. These improvements were accompanied by enhanced writing motivation, increased English writing self-efficacy and self-concept while students' English writing apprehension and anxiety decreased. The results support the conclusion that using genre-based instruction develops increased language awareness and writing competence, thereby motivating students to write.

dos Santos Silva et al.: *Attitudes in music practice: a survey exploring the self-regulated learning processes of advanced Brazilian and Portuguese musicians*

Three articles in this Research Topic focus on self-regulated learning (SRL). In the first of the three, dos Santos Silva et al., investigated learning behaviors vary among advanced musicians based on factors such as gender, nationality, instrument, practice quantity, expertise, and professional experience. The authors utilized a 22-item questionnaire completed by 300 participants, to identify three key SRL components: Practice Organization, Personal Resources, and External Resources. The results indicate that as musicians gain experience, their metacognitive processes become more prominent than the social factors influencing their performance. Furthermore, the study suggests that SRL processes are acquired throughout a musician's learning journey and become internalized with significant practice, leading to more efficient practice sessions and reduced time to achieve performance goals. As musicians reach higher levels of professional performance, personal resources tend to surpass reliance on external factors. This study provides important insights into SRL that are helpful for the understanding of two other studies in this Research Topic, those of López-Íñiguez and McPherson as well as Pucihar et al. and reaffirms the fact that, ideally, all institutions of music education would invest in teacher education. Because as important as SRL is for musicians, theoretical knowledge has been slow to be implemented into practice in some countries. Ideally, all institutions of music education would invest in teacher SRL education, support a focus on quality of practice and metacognitive strategies over efficiency, and encourage students to improve self-regulation.

Pucihar et al.: *The key reasons for dropout in Slovenian music schools – a qualitative study*

Pucihar et al., also employed self-determination theory, in this case to examine the difficulties students encounter in sustaining long-term commitment to and motivation for music studies. This study identified external factors such as ineffective teaching approaches, classroom social environment, problematic curricular matters, and limited resources as well as internal issues including a lack of perceived autonomy (student choice), feelings of competence (underdeveloped musical skills), and deficiencies in relatedness (with teachers and/or peers) as contributors leading to student dropout. Their study suggests that a holistic, individualized approach that allows a nurturing learning environment to promote autonomy, competence and relatedness can address these multiple concerns. Implementing the findings of this study could enhance the musical education of participants and reduce dropout rates of students.

## Conclusions

Implications of these articles on motivation in learning and performance include recommendations for taking these results into practice. First, create supportive learning environments. For example, by providing learners with choices, opportunities for skill development, and a sense of belonging. Second, encourage adaptive emotion regulation strategies, such as cognitive reappraisal, to better manage emotions in challenging learning situations. Teach learners how to reframe their thoughts and perceptions of difficult tasks, and provide them with opportunities to practice these strategies. Third, create a mastery-oriented training climate to foster intrinsic motivation, enjoyment, and a willingness to embrace challenges rather than a singular focus on outcomes and comparisons with others. Finally, promote positive emotions and adaptive emotion regulation, to enhance both performance and wellbeing.
