# Correction: Editorial: Motivation in learning and performance in the arts and sports

**DOI:** 10.3389/fpsyg.2025.1699580

**Published:** 2025-10-16

**Authors:** Adina Mornell, Margaret S. Osborne, Noa Kageyama, Frank Heuser

**Affiliations:** ^1^University of Music and Theater Munich, Munich, Germany; ^2^Melbourne School of Psychological Sciences and Melbourne Conservatorium of Music, University of Melbourne, Melbourne, VIC, Australia; ^3^The Juilliard School, New York, NY, United States; ^4^Cleveland Institute of Music, Cleveland, OH, United States; ^5^The UCLA Herb Alpert School of Music, University of California, Los Angeles, Los Angeles, CA, United States

**Keywords:** motivation, music, performance science, pedagogy, cognitive reappraisal, wellbeing

In the published article, there was an error. The editorial has a mistake: It does not accurately describe the dos Santos Silva et al. submission entitles “Attitudes in music practice: a survey exploring the self-regulated learning processes of advanced Brazilian and Portuguese musicians.”

A correction has been made to **[Editorial]**, *Sub-Section* “**dos Santos Silva, Araújo & Marinho: Attitudes in music practice: a survey exploring the self-regulated learning processes of advanced Brazilian and Portuguese musicians”** on page 10 of the editorial. This paragraph previously stated:

“Three articles in this Research Topic focus on self-reflective learning (SRL). The first, of the three, a systematic review by **dos Santos Silva et al.**, provides the groundwork for an understanding of SRL that is important to two other studies in this collection, those of López-Íñiguez and McPherson as well as Pucihar et al. Dos santos Silva et al. used the “Preferred Reporting Items for Systematic Reviews and Meta-Analyses” or PRISMA protocol to evaluate scientific literature on SRL employing the current “gold standard” for such meta reviews. Indeed, this paper can serve as instructional for those wishing to engage in such analyses. The background section of this paper lays out the theoretical development of SRL with precision and valuable citations both of historical significance and current relevance. What also makes their study especially important for the world-wide community of researchers is the fact that the authors considered papers in four languages: English, Spanish, Portuguese, and French. The authors suggest in their conclusion, that in these cultures, too much time in both music teaching and practice still adheres to tradition and propagates old habits. As important as SRL may be for goal-setting, such theoretical knowledge is slow to be implemented into practice in some countries. Ideally, all institutions of music education would invest in teacher SRL education, support a focus on quality of practice and metacognitive strategies over efficiency, and encourage students to improve self-regulation.”

The corrected paragraph appears below:

“Three articles in this Research Topic focus on self-regulated learning (SRL). In the first of the three, dos Santos Silva et al., investigated learning behaviors vary among advanced musicians based on factors such as gender, nationality, instrument, practice quantity, expertise, and professional experience. The authors utilized a 22-item questionnaire completed by 300 participants, to identify three key SRL components: Practice Organization, Personal Resources, and External Resources. The results indicate that as musicians gain experience, their metacognitive processes become more prominent than the social factors influencing their performance. Furthermore, the study suggests that SRL processes are acquired throughout a musician's learning journey and become internalized with significant practice, leading to more efficient practice sessions and reduced time to achieve performance goals. As musicians reach higher levels of professional performance, personal resources tend to surpass reliance on external factors. This study provides important insights into SRL that are helpful for the understanding of two other studies in this Research Topic, those of López-Íñiguez and McPherson as well as Pucihar et al. and reaffirms the fact that, ideally, all institutions of music education would invest in teacher education. Because as important as SRL is for musicians, theoretical knowledge has been slow to be implemented into practice in some countries. Ideally, all institutions of music education would invest in teacher SRL education, support a focus on quality of practice and metacognitive strategies over efficiency, and encourage students to improve self-regulation.”

The original version of this article has been updated.

